# Interaction of Lipoplex with Albumin Enhances Gene Expression in Hepatitis Mice

**DOI:** 10.3390/pharmaceutics12040341

**Published:** 2020-04-10

**Authors:** Naoki Yoshikawa, Shintaro Fumoto, Keiko Yoshikawa, Die Hu, Kazuya Okami, Riku Kato, Mikiro Nakashima, Hirotaka Miyamoto, Koyo Nishida

**Affiliations:** 1 Department of Pharmacy, University of Miyazaki Hospital, 5200 Kihara, Kiyotake-cho, Miyazaki 889-1692, Japan; 2 Graduate School of Biomedical Sciences, Nagasaki University, 1-7-1 Sakamoto, Nagasaki-shi, Nagasaki 852-8501, Japan

**Keywords:** in vivo gene transfection, lipofection, hepatitis, interaction with serum components, lung, liver

## Abstract

Understanding the in vivo fate of lipoplex, which is composed of cationic liposomes and DNA, is an important issue toward gene therapy. In disease conditions, the fate of lipoplex might change compared with the normal condition. Here, we examined the contribution of interaction with serum components to in vivo transfection using lipoplex in hepatitis mice. Prior to administration, lipoplex was incubated with serum or albumin. In the liver, the interaction with albumin enhanced gene expression in hepatitis mice, while in the lung, the interaction with serum or albumin enhanced it. In normal mice, the interaction with albumin did not enhance hepatic and pulmonary gene expression. Furthermore, hepatic and pulmonary gene expression levels of albumin-interacted lipoplex were correlated with serum transaminases in hepatitis mice. The albumin interaction increased the hepatic accumulation of lipoplex and serum tumor necrosis factor-α level. We suggest that the interaction with albumin enhanced the inflammation level after the administration of lipoplex in hepatitis mice. Consequently, the enhancement of the inflammation level might enhance the gene expression level. Information obtained in the current study will be valuable toward future clinical application of the lipoplex.

## 1. Introduction

Gene therapy is a good choice for refractory diseases. Recent progress in gene technologies has improved the possibility of success of gene therapy. Among the refractory diseases, late-stage hepatic disease is a critical target of gene therapy because of the few therapeutic methods that can provide complete therapy.

In order to succeed in gene therapy, gene medicines have been developed. Moreover, gene vectors are expected as effective gene medicine. Gene vectors are broadly classified into viral [1,2] and non-viral types [3,4,5,6]. Plasmid DNA (pDNA) is one of the typical non-viral gene vectors, but naked pDNA itself does not show sufficient transfection efficiency after intravenous injection with normal volume. Therefore, cationic liposome-pDNA complex (lipoplex) is often used in transfection [7,8]. The surface of lipoplex is positively charged because of cationic liposomes. Thus, lipoplex would interact with biological components such as erythrocytes and plasma proteins, which possess negative surface charge. Especially, protein corona, defined as protein-wrapping on the surface of nanoparticles, is known as an important factor for liposome and lipoplex usages [9,10]. It is known that the presence of serum in culture media inhibits in vitro transfection in cell culture [11]. In contrast, pre-incubation of lipoplex with serum did not decrease gene expression in the lung after intravenous (i.v.) injection, whereas pre-incubation with red blood cells did [12]. In the case of intraportal injection (i.p.), serum enhanced gene expression of galactosylated lipoplex in the liver [13]. Furthermore, we reported that multiple components including calcium ion and fibronectin contributed to gene expression in the liver after i.p. injection of unmodified lipoplex [14]. Moreover, calcium ion and fibronectin also contributed to pulmonary gene expression after i.v. injection [15].

For applying lipoplex in a clinical setting, analysis for the interaction of lipoplex with biological components in disease states is needed. Yoshioka et al. reported that hepatitis disease stages affected the gene expression level of lipoplex after i.v. injection in carbon tetrachloride (CCl_4_)-induced hepatitis mice [16]. Hepatitis might change the gene transfection process, such as the disposition of lipoplex and its cellular uptake, the cytoplasmic release of pDNA, and nuclear translocation of pDNA. Furthermore, hepatitis can also change the level of serum proteins and may influence the transfection after intravascular injection. Actually, the synthesis of plasma proteins, such as albumin, fibrinogen, and gamma globulin, was decreased in CCl_4_-induced hepatitis rat [17].

The liver is an important organ to perform metabolism and excretion. Hepatic diseases such as acute and chronic hepatitis, fibrosis, cancer, and inheritable gene deficiency diseases are targets of gene therapy. In addition, hepatic disease is often accompanied by non-hepatic diseases. The application of gene therapy in these hepatic diseases is extremely important in order to repair biological functions. Therefore, in this study, we analyzed the contribution of serum components to in vivo gene transfer using lipoplex in CCl_4_-induced hepatitis mice.

## 2. Materials and Methods 

### 2.1. Materials

1,2-Dioleoyl-3-trimethylammonium-propane methyl sulfate salt (DOTAP) and 1,2-dioleoyl-*sn*-glycero-3-phosphoethanolamine-*N*-(lissamine rhodamine B sulfonyl) (Rh-DOPE) were obtained from Avanti Polar Lipids, Inc. (Alabaster, AL, USA). All chemicals were of the highest purity available.

### 2.2. Animals

Male ddY mice (5 weeks old, weighing 24.0–33.4 g) were purchased from Kyudo Co., Ltd. (Kumamoto, Japan). They were housed in a cage in an air-conditioned room and maintained on a standard laboratory diet (MF, Oriental Yeast, Co., Ltd., Tokyo, Japan) and water ad libitum. All animal experiments were carried out under the Guidelines for Animal Experimentation of Nagasaki University (Approval number, 1304171055; Approval Date, 17 April, 2013).

### 2.3. pDNA

Firefly luciferase-encoding pDNA pCMV-luciferase was constructed previously [15]. pDNA was isolated from *E. coli* strain DH5α and purified using an EndoFree^®^ Plasmid Giga Kit (QIAGEN GmbH, Hilden, Germany).

### 2.4. Cationic Liposomes

Cationic liposomes were prepared as reported previously [13,15,18,19] based on thin lipid film hydration method. Liposomes are composed of DOTAP and cholesterol at a molar ratio of 1:1. The dispersion medium was sterile 5% glucose solution (4 mg/mL). Liposomes were extruded 11 times using a Mini-Extruder with 100 nm filter (Avanti Polar Lipids, Inc.). Rhodamine-labeled liposomes containing Rh-DOPE (0.25 mol% of the total lipids) were prepared in the same fashion.

### 2.5. Lipoplex

pDNA in 5% glucose solution (100 μL of 300 μg/mL) was mixed with liposomes (100 μL of 3170 μg/mL and the charge ratio (+/−) of 3.0), then incubated for 30 min at 37 °C to form lipoplex [15]. Particle size and zeta potential of lipoplex were measured using a Zetasizer Nano ZS instrument (Malvern Instruments Ltd., Worcestershire, UK).

### 2.6. Preparation of Serum Components

Bovine serum albumin (BSA) was purchased from Sigma Chemicals Inc. (St. Louis, MO, USA). The concentration of BSA was 30 mg/mL [20] in phosphate-buffered saline (PBS (-)). To obtain serum, mouse blood was collected from anesthetized mice, clotted and centrifuged at 15,000 × *g* for 5 min. As control experiments, lipoplex was incubated with 5% glucose solution to adjust injection volume.

### 2.7. In Vivo Transfection

Prior to injection, lipoplex containing 30 μg of pDNA in 200 μL was incubated with serum components in 100 μL for 5 min at 37 °C. The mixture of lipoplex and serum components (30 μg pDNA/300 μL) was injected via a tail vein in mice. Luciferase activity in tissues homogenate supernatant was determined after tissue homogenization with a lysis buffer (4 μL/mg tissue, 0.1 M Tris/HCl buffer (pH 7.8) containing 0.05% Triton X-100 and 2 mM EDTA [15,18,21]) and centrifugation, in the same fashion to a previous report [15]. The luciferase activity is indicated as the relative light units (RLU) per gram of tissue.

### 2.8. Measurement of Serum Transaminase Activities

Serum transaminase activities were determined using a Transaminase C-II test Wako kit (Wako Pure Chemical Industries, Ltd., Osaka, Japan) [22].

### 2.9. Evaluation of Hepatic Accumulation of Lipoplex

The accumulated amount of rhodamine-labeled lipoplex in tissues was evaluated by determining the fluorescent intensity of Rh-DOPE extracted from tissues, as previously reported [15,23]. Rhodamine-labeled lipoplex was injected via a tail vein in mice. Two minutes after administration, blood samples were collected and the mice were killed under anesthesia. The liver was removed with surgical scissors, washed with saline, and homogenized with PBS (-). To extract Rh-DOPE, 0.175 mL of homogenate or blood sample was added to 0.420 mL of methanol, 0.438 mL of chloroform and 0.175 mL of saturated aqueous sodium chloride, and then the mixture was vigorously shaken for 5 min, followed by centrifugation at 15,000× *g* for 10 min. The amount of Rh-DOPE in the lower layer was evaluated by determining the fluorescence intensity at excitation and emission wavelengths of 544 and 590 nm using spectrofluorometer RF-1500 (Shimadzu Corp., Kyoto, Japan).

### 2.10. Cytokine ELISA

Tumor necrosis factor (TNF)-α in serum was measured by enzyme-linked immunosorbent assay (ELISA) with the ELISA Ready-SET-Go! Kit (eBioscience, San Diego, CA, USA).

### 2.11. CCl_4_-Induced Hepatitis Mice

Hepatitis mice were prepared by intraperitoneal injection of CCl_4_ in olive oil (1%, 10 µL/g mice) [22,24,25]. Twenty-four hours after the injection, lipoplex was administered.

### 2.12. Statistical Analysis

Statistical comparisons were performed by a Student’s *t*-test for two groups or by a Dunnett’s test for multiple comparisons with a control group. The relationship between gene expression level and serum transaminase activity level was analyzed by Pearson’s product–moment correlation coefficient.

## 3. Results

### 3.1. Effect of the Interaction with Serum Components on Gene Expression in Hepatitis Mice

**Figure 1:**
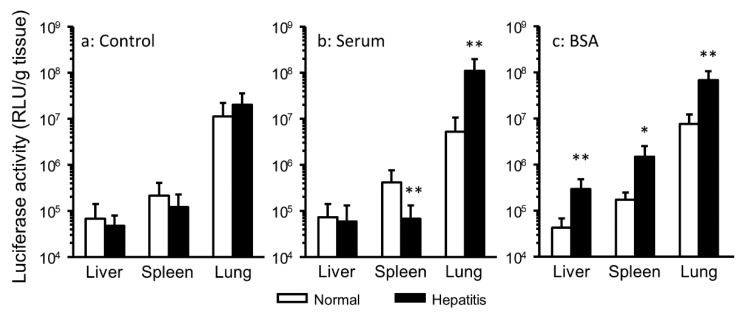
Effect of hepatitis on gene expression in each tissue after i.v. injection. Lipoplex was incubated with 5% glucose (control, **a**), serum (**b**), or BSA (**c**). Each bar represents the mean + SD of at least 6 experiments. Statistical method, Student’s *t*-test. * *p* < 0.05, ** *p* < 0.01 vs. normal.

**Figure 2:**
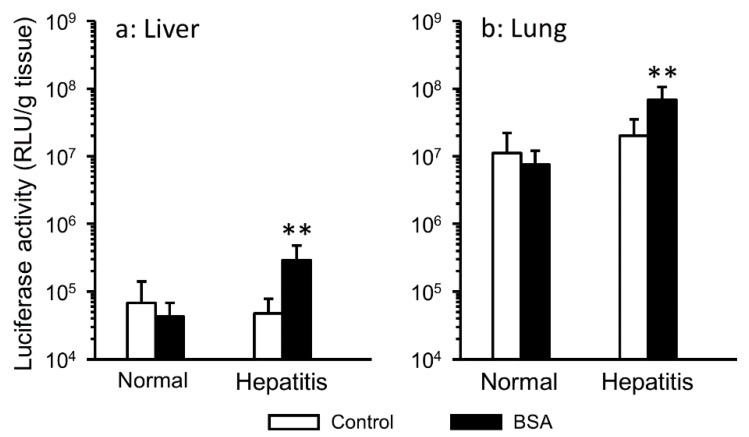
Effect of interaction of lipoplex with albumin on gene expression in the liver (**a**) and lung (**b**) after i.v. injection of lipoplex in normal or hepatitis mice. Lipoplex was incubated with 5% glucose (control) or BSA. Each bar represents the mean + SD of at least 6 experiments. Statistical method, Student’s *t*-test. ** *p* < 0.01 vs. control.

Serum aminotransferase activities (ALT and AST) serve as parameters to evaluate liver disease in clinical practice. It was reported that serum aminotransferases peaked 24 h after CCl_4_ administration [26]. Thus, lipoplex interacted with serum components was injected 24 h after CCl_4_ treatment in mice to analyze the effect of serum components on gene transfer in hepatitis mice (Figure 1). In the serum group, hepatitis significantly enhanced pulmonary gene expression (Figure 1B). In the BSA group, hepatitis significantly enhanced gene expression in all harvested tissue (Figure 1C). Generally, pulmonary gene expression is highest after i.v. injection of cationic lipoplex. Hence, we focused on gene expression in the liver, which is inflamed tissue, and the lung (Figure 2). In hepatitis mice, the interaction with BSA significantly enhanced hepatic and pulmonary gene expression, but in normal mice, it did not.

### 3.2. Effect of the Interaction with Serum Components on Serum Transaminase Activities

**Figure 3:**
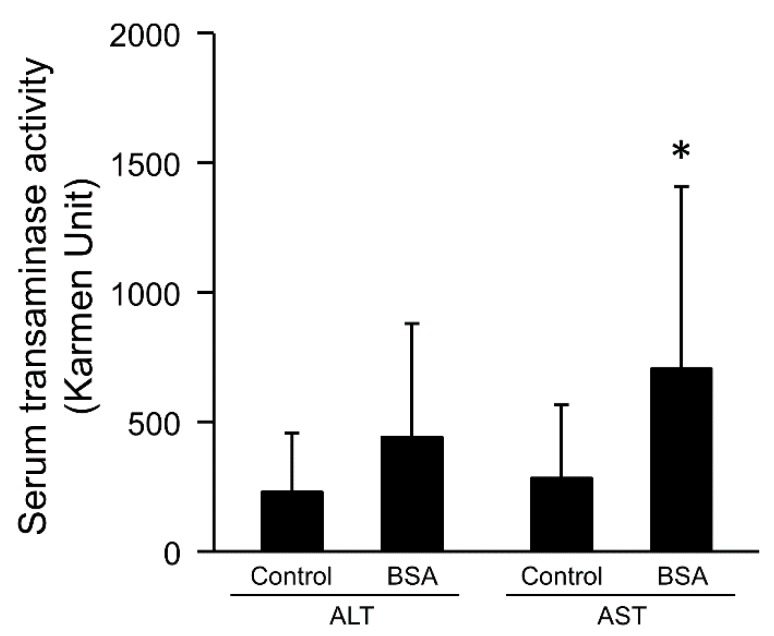
Effect of interaction of lipoplex with albumin on serum transaminase activities 6 h after i.v. injection in hepatitis mice. Lipoplex was incubated with 5% glucose (control) or BSA. Each bar represents the mean + SD of at least 6 experiments. Statistical method, Student’s *t*-test. * *p* < 0.05 vs. control.

It is known that AST is contained not only in the liver, but also in other tissues such as cardiac muscles, skeletal muscles, brain, kidneys, and leukocytes. Although ALT is contained in some tissue, like AST, its level in liver is higher than in other tissues. The administration of CCl_4_ increased both ALT and AST [22,27]. AST activity was significantly increased by the interaction of lipoplex with BSA in hepatitis mice, while BSA increased ALT activity slightly (Figure 3).

### 3.3. Correlation between Gene Expression Levels and Serum Transaminase Activity Levels in Hepatitis Mice

**Figure 4:**
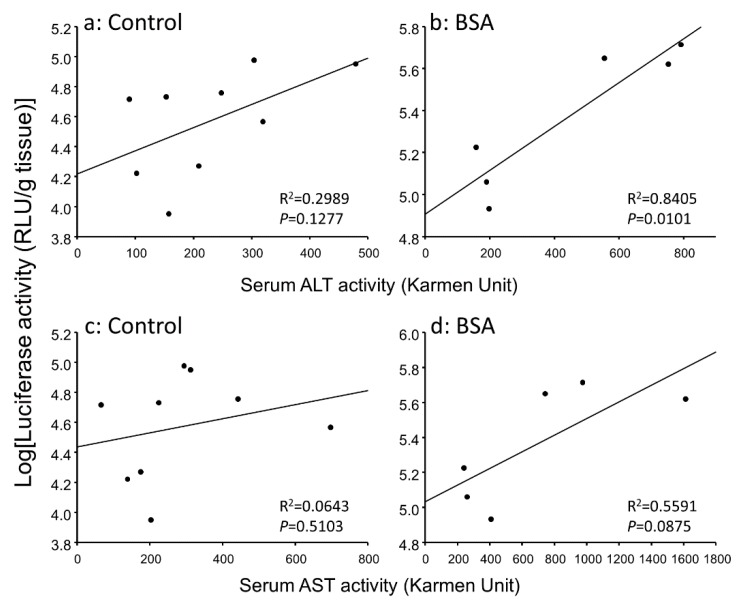
Relationship between hepatic gene expression level and serum ALT (**a**,**b**) or AST (**c**,**d**) activity level 6 h after i.v. injection of lipoplex incubated with 5% glucose (**a**,**c**) or BSA (**b**,**d**) in hepatitis mice. Solid line represents the regression curve.

**Figure 5:**
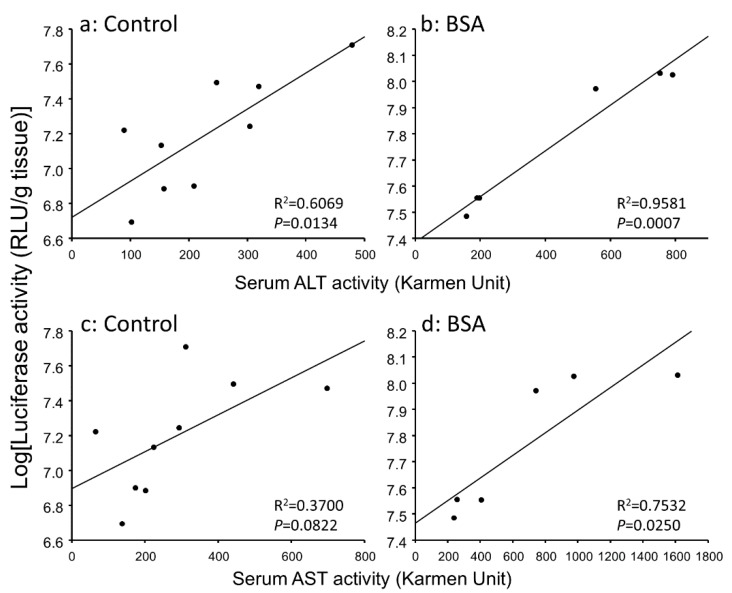
Relationship between pulmonary gene expression level and serum ALT (**a**,**b**) or AST (**c**,**d**) activity level 6 h after i.v. injection of lipoplex incubated with 5% glucose (**a**,**c**) or BSA (**b**,**d**) in hepatitis mice. Solid line represents the regression curve.

In hepatitis mice, the inflammation reaction might contribute to the enhanced gene expression. Hence, we analyzed the relationship between gene expressions and serum transaminase activities. Figure 4 and Figure 5 show the correlation between hepatic and pulmonary gene expression levels and serum transaminase activity level, respectively. Hepatic gene expression level was significantly correlated with serum ALT activity level in the BSA group (Figure 4B). Pulmonary gene expression level was better correlated with serum ALT and AST activity levels than hepatic gene expression level (Figure 5). Thus, the inflammation level in the liver could explain enhanced gene expression not only in the liver but also in the lung.

### 3.4. Changes in Hepatic Accumulation of Lipoplex by Interaction with Albumin in Hepatitis Mice

**Figure 6:**
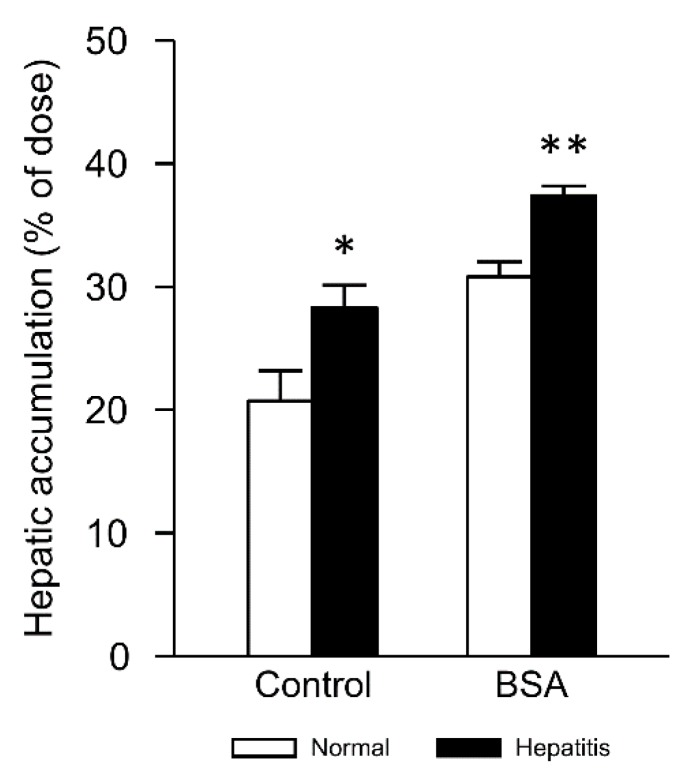
Effect of hepatitis on hepatic accumulation of lipoplex after i.v. injection in mice. Lipoplex was incubated with 5% glucose (control) or BSA for 5 min before injection. Each bar represents the mean + SD of 3 experiments. Statistical method, Student’s *t*-test. * *p* < 0.05, ** *p* < 0.01 vs. normal.

Next, we evaluated the hepatic accumulation of lipoplex 2 min after i.v. injection. Hepatitis significantly increased the hepatic accumulation in control and BSA groups (Figure 6). Furthermore, incubation of lipoplex with BSA tended to increase hepatic accumulation compared with control groups. Therefore, increased hepatic accumulation may explain the increased inflammation level in the liver. 

### 3.5. Effect of the Interaction with Albumin on Serum TNF-α Level

**Figure 7:**
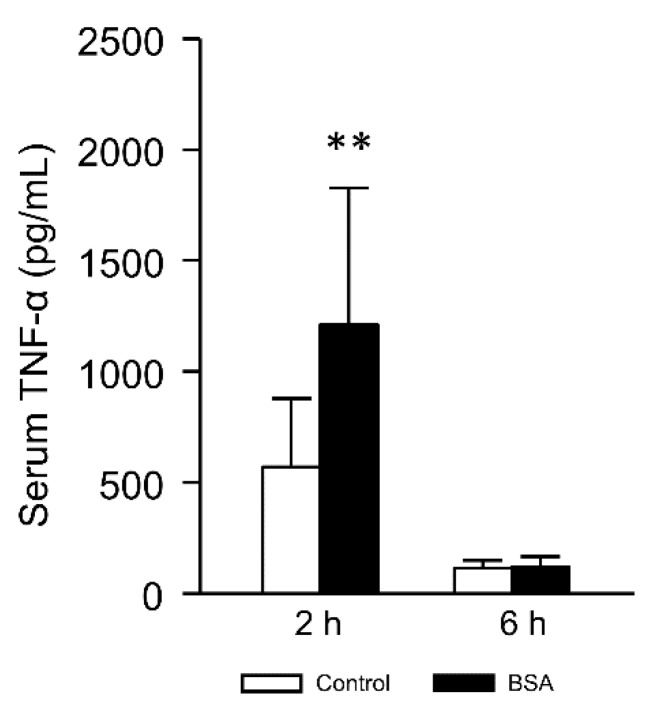
Effect of albumin interaction on serum TNF-α 2 h and 6 h after i.v. injection of lipoplex in hepatitis mice. Lipoplex was incubated with 5% glucose (control) or BSA. Each bar represents the mean + SD of at least 8 experiments. Statistical method, Student’s *t*-test. ** *p* < 0.01 vs. control.

Serum levels of TNF-α and interleukin-1β were significantly increased after the administration of CCl_4_ in rats and mice [28,29]. Furthermore, the administration of lipoplex induced several inflammatory cytokines [30]. The cytokines are important indices of the inflammation level. Thus, to evaluate the effect of BSA interaction on the inflammation level, we measured the serum TNF-α level after lipoplex injection. Figure 7 shows the serum TNF-α level 2 h and 6 h after the injection in hepatitis mice. BSA interaction significantly enhanced TNF-α level 2 h after the injection. Increased TNF-α level in BSA group could be explained by increased hepatic accumulation (Figure 6).

### 3.6. Effect of pH on Particle Size and Zeta Potential of Lipoplex Interacted with BSA

Under the hepatitis condition, pH in the liver decreased from the physiological value of 7.4. Then, we tested the effect of pH on the size (Table 1) and zeta potential (Table 2) of lipoplex to consider the effect of physicochemical properties on increased hepatic accumulation. Incubation of lipoplex with PBS (-) increased the particle size twice compared with that of 5% glucose (222.5 nm). Decreasing pH from 7.4 to 5.5 decreased the size of control lipoplex, while the size of lipoplex interacted with BSA did not change by pH. Zeta potential of both control and BSA-interacted lipoplexes increased by decrease of pH. Increased zeta poteintial might explain the increased hepatic accumulation in hepatitis mice (Figure 6).

## 4. Discussion

Many groups research gene therapy to cure liver diseases. For instance, hepatocyte growth factor (HGF), which is one of the candidate therapeutic genes, is expected to regenerate hepatic tissue from fibrotic tissue. Although lipoplex was investigated in many reported studies, reports of the effects of biological factors on the transfection efficiency of lipoplex were limited. Therefore, it is important to analyze the interaction between lipoplex and serum components in hepatitis in order to succeed in hepatic gene therapy.

In the present study, the gene expression level after i.v. injection of lipoplex interacted with serum or BSA in hepatitis mice differed from that in normal mice (Figure 1). It is considered that various biological changes occur in hepatitis mice. It was reported that the susceptibility to pneumococcal pneumonia increased in CCl_4_-induced liver cirrhosis model rats [31]. Additionally, fulminant hepatitis patients occasionally had pulmonary edema [32]. Hence, it was considered that the inflammation both in the liver and in the lung was caused by CCl_4_ in the present study. In inflammatory tissue, the intracellular and tissue pH is lower than that in the normal condition [33,34]. The administration of CCl_4_ also decreased protein synthesis by the liver [17]. These changes of the biological environment might affect gene transfer, such as the biodistribution and cellular uptake of lipoplex in hepatitis mice. Additionally, the interaction with serum components was one of the factors involved in enhancing gene expression (Figure 2). In particular, the interaction with albumin, which is a major protein in serum, played a critical role in hepatic gene expression. It was thought that the role of albumin interacted with lipoplex differed between normal tissues and inflammatory tissues.

It is known that serum transaminases are increased by hepatocyte necrosis. Therefore, serum transaminases are increased by not only hepatic disorder but also administration of lipoplex [30,35]. In this study, the interaction with albumin increased the serum AST activity level after i.v. injection in hepatitis mice (Figure 3). Due to the fact that the interaction with albumin enhanced the gene expression level and the inflammation level after i.v. injection of lipoplex, we analyzed the relationship between the gene expression and inflammation. Hepatic and pulmonary gene expression levels were correlated with serum ALT and AST activity levels in hepatitis mice (Figure 4 and Figure 5), suggesting that induction of hepatic inflammation might have a relationship to the enhancement of gene expression. The interaction with BSA produced more significant correlation.

Increase of the cellular uptake of lipoplex was considered as a factor that enhanced gene expression and inflammation level after i.v. injection. Actually, we showed that hepatitis increased the hepatic accumulation of lipoplex interacted with BSA (Figure 6). It was reported that albumin uptake was enhanced in tumor tissues [36]. It is known that tumor tissues, similar to inflammatory tissues, show lower pH than normal tissues [37,38]. This suggests that enhanced hepatic accumulation of lipoplex interacted with albumin might be attributed to lower pH in the liver of hepatitis mice than that in normal mice.

Many researchers reported that liposomes were opsonized by interaction with serum, and the opsonized liposomes were ingested by the reticuloendothelial system [39,40]. However, the function of Kupffer cells and the uptake of *Streptococcus pneumoniae* by polymorphonuclear leukocytes decreased in CCl_4_-induced cirrhosis rats [31,41]. Furthermore, phagocytic activity of the reticuloendothelial system was suppressed in CCl_4_-induced liver injury mice [42]. Therefore, it was considered that phagocytosis of lipoplex was decreased in the reticuloendothelial system after CCl_4_ treatment. On the other hand, inflammatory cytokine release from Kupffer cells is activated by CCl_4_ treatment [28,43]. It was also reported that Kupffer cells and TNF-α were involved in liver toxicity caused by CCl_4_ [44,45]. Moreover, TNF-α activates transcriptional factor NF-κB [46,47,48]. pDNA used in the present study has many NF-κB binding sites, suggesting that gene expression might be enhanced by NF-κB activation, similar to a previous report [49]. TNF-α was increased by the interaction of lipoplex with BSA in hepatitis mice (Figure 7). It was possible that the interaction with BSA affected inflammatory cytokine release from Kupffer cells by changes in the recognition of lipoplex by Kupffer cells.

About physicochemical properties, we have reported the changes in size and zeta potential of lipoplex after incubation with serum components [14]. About particle size, diameter of control lipoplex was ca. 230 nm [14]. Incubation with serum increased the size 2.24 times, while incubation with BSA did not significantly change it (1.06 times) [14]. After incubation with serum components, zeta potential of lipoplex became a negative charge [14]. The effect of these changes in physicochemical properties on in vivo transfection might be complicated. In spite of the charge reversal, pulmonary accumulation of lipoplex after incubation of serum did not change in normal mice [15]. In the case of serum, we have clarified the presence of fibronectin in serum contributed to pulmonary accumulation and gene expression [15]. In this study, incubation with BSA increased hepatic accumulation, especially in hepatitis mice (Figure 6). In the case of BSA, different from serum, there seemed to be no specific components like fibronectin to increase hepatic accumulation and gene expression. About control lipoplex, the size should be increased due to interaction with blood components after intravenous injection. Therefore, penetration through fenestrated endothelium in the liver might be restricted by their size. On the other hand, interaction with BSA prior to injection might inhibit such an increase after intravenous injection, subsequently more penetration might occur. In CCl_4_-induced hepatitis condition, extracellular pH in the liver might decrease, as is similar with the case of acute liver ischemia/reperfusion injury [50]. Under the decreased pH condition, zeta potential of the lipoplex increased (Table 2). This might explain increased hepatic accumulation of the lipoplex in hepatitis mice because increased surface charge could enhance the interaction of lipoplex with negatively charged cells. Furthermore, the initial inflammation level at the timing of lipoplex injection might affect hepatic accumulation of lipoplex based on a pH-dependent manner. The increased hepatic accumulation could increase the release of inflammatory cytokines via the CpG motif-dependent manner. Then, this might worsen hepatic inflammation and increase gene expression.

## 5. Conclusions

We found that hepatic and pulmonary gene expressions after i.v. injection of lipoplex interacted with albumin were enhanced in CCl_4_-induced hepatitis mice. Furthermore, we elucidated that the gene expression level correlated to the inflammation level. We considered that the increase of hepatic accumulation of lipoplex and TNF-α were important factors enhancing gene expression. The effects of serum components on in vivo gene transfer changed in hepatitis mice.

## Figures and Tables

**Table 1:** Changes in particle size of lipoplex by pH.

Lipoplex	pH 7.4	pH 7.0	pH 6.5	pH 6.0	pH 5.5
Control	473 ± 24.5	491 ± 33.3	423 ± 20.2	437 ± 35.6	389 ± 21.9 *
BSA-interacted	208 ± 5.63	207 ± 7.60	204 ± 7.50	200 ± 1.22	203 ± 5.03

Lipoplex was incubated with PBS (-), of which pH was adjusted to indicated values. Unit, nm. Each value represents the mean ± SD of 3 experiments. Statistical method, Dunnett’s test. * *p* < 0.05 vs. pH 7.4.

**Table 2:** Changes in zeta potential of lipoplex by pH.

Lipoplex	pH 7.4	pH 7.0	pH 6.5	pH 6.0	pH 5.5
Control	39.7 ± 2.41	43.5 ± 1.25 *	44.9 ± 0.17 **	47.6 ± 1.65 **	48.3 ± 1.04 **
BSA-interacted	−11.7 ± 0.76	−10.5 ± 0.85	−9.50 ± 0.35 *	−8.21 ± 0.82 **	−7.36 ± 0.66 **

Lipoplex was incubated with PBS (-), of which pH was adjusted to indicated values. Unit, mV. Each value represents the mean ± SD of 3 experiments. Statistical method, Dunnett’s test. * *p* < 0.05, ** *p* <0.01 vs. pH 7.4.
